# Deciphering a shared transcriptomic regulation and the relative contribution of each regulator type through endometrial gene expression signatures

**DOI:** 10.1186/s12958-023-01131-4

**Published:** 2023-09-12

**Authors:** Antonio Parraga-Leo, Patricia Sebastian-Leon, Almudena Devesa-Peiro, Diana Marti-Garcia, Nuria Pellicer, Jose Remohi, Francisco Dominguez, Patricia Diaz-Gimeno

**Affiliations:** 1 IVIRMA Global Research Alliance, IVI Foundation, Instituto de Investigación Sanitaria La Fe (IIS La Fe), Av. Fernando Abril Martorell 106, Torre A, Planta 1ª, 46026 Valencia, Valencia Spain; 2 Department of Pediatrics, Obstetrics and Gynaecology, Universidad de Valencia, Av. Blasco Ibáñez 15, 46010 Valencia, Valencia Spain; 3 IVIRMA Global Research Alliance, IVIRMA Valencia, Plaza de La Policia Local 3, 46015 Valencia, Spain

**Keywords:** *CTCF*, GATA6, Progesterone, Estrogen, miRNAs, TFs, Endometrial receptivity, Recurrent implantation failure, Infertility, Menstrual cycle regulation

## Abstract

**Backgorund:**

While various endometrial biomarkers have been characterized at the transcriptomic and functional level, there is generally a poor overlap among studies, making it unclear to what extent their upstream regulators (e.g., ovarian hormones, transcription factors (TFs) and microRNAs (miRNAs)) realistically contribute to menstrual cycle progression and function. Unmasking the intricacies of the molecular interactions in the endometrium from a novel systemic point of view will help gain a more accurate perspective of endometrial regulation and a better explanation the molecular etiology of endometrial-factor infertility.

**Methods:**

An *in-silico* analysis was carried out to identify which regulators consistently target the gene biomarkers proposed in studies related to endometrial progression and implantation failure (19 gene lists/signatures were included). The roles of these regulators, and of genes related to progesterone and estrogens, were then analysed in transcriptomic datasets compiled from samples collected throughout the menstrual cycle (*n* = 129), and the expression of selected TFs were prospectively validated in an independent cohort of healthy participants (*n* = 19).

**Results:**

A total of 3,608 distinct genes from the 19 gene lists were associated with endometrial progression and implantation failure. The lists’ regulation was significantly favoured by TFs (89% (17/19) of gene lists) and progesterone (47% (8 /19) of gene lists), rather than miRNAs (5% (1/19) of gene lists) or estrogen (0% (0/19) of gene lists), respectively (FDR < 0.05). Exceptionally, two gene lists that were previously associated with implantation failure and unexplained infertility were less hormone-dependent, but primarily regulated by estrogen. Although endometrial progression genes were mainly targeted by hormones rather than non-hormonal contributors (odds ratio = 91.94, FDR < 0.05), we identified 311 TFs and 595 miRNAs not previously associated with ovarian hormones. We highlight *CTCF*, GATA6, hsa-miR-15a-5p, hsa-miR-218-5p, hsa-miR-107, hsa-miR-103a-3p, and hsa-miR-128-3p, as overlapping novel master regulators of endometrial function. The gene expression changes of selected regulators throughout the menstrual cycle (FDR < 0.05), dually validated *in-silico* and through endometrial biopsies, corroborated their potential regulatory roles in the endometrium.

**Conclusions:**

This study revealed novel hormonal and non-hormonal regulators and their relative contributions to endometrial progression and pathology, providing new leads for the potential causes of endometrial-factor infertility.

**Supplementary Information:**

The online version contains supplementary material available at 10.1186/s12958-023-01131-4.

## Background

The endometrium is the innermost layer of the uterus, which undergoes dynamic histological, physiological and molecular changes that allow it to synchronize with embryo development, facilitate embryo implantation, and ultimately, establish a successful pregnancy [1].

Implantation is a crucial and complex limiting step for conception [2] which occurs between days 19 to 24 of a normal menstrual cycle. Consequently, this period of time is called the window of implantation (WOI) [3], and is characterized by abrupt transcriptomic changes in the endometrial tissue [4]. Alterations in either WOI establishment and endometrial progression may lead to implantation failures, which, along with biochemical miscarriage, account for more than 50% of pregnancy losses at pre-clinical stages [5, 6]. These alterations have broadly been classified as being related to a displaced WOI, caused by variable timing of endometrial progression, or a disrupted WOI, where impaired endometrial function prevents the establishment of an effective WOI [7].

Initially, the WOI was postulated to mainly be regulated by the ovarian hormones (namely, estrogen and progesterone) acting via their respective nuclear receptors (i.e., *ESR1/2* and *PGR*). However, evidence has shown that these receptors cooperate with other transcription factors (TFs) and co-regulators to mediate uterine physiology [8]. In fact, TFs (such as homeobox TFs, *FOXA2,* and *KLF9)* have recently been reported as key regulators for the establishment of endometrial receptivity [9, 10], and microRNAs (miRNAs) have been found to similarly intervene in this complex and multifactorial process as transcriptional regulators [11]. While the involvement of both types of regulators was widely reported in embryo implantation [12] and other infertility-related diseases, including recurrent implantation failure (RIF) [13], the complex interactions between the different types of regulators and their respective contributions to endometrial progression and function have not been investigated from a systemic point of view. This paradigm shift approaches genes and regulators in an integrative way, by considering how they interact with and coordinate each other to carry out cellular processes, rather than reinforcing the premise they work independently [14]. By more accurately reflecting the biological milieu, systemic approaches take gene-based discoveries to the next level, helping to generate hypotheses with relevant clinical and molecular implications [15, 16].

Previous studies of endometrial transcriptomics have been used to define the WOI, compare the endometrium of healthy women as their menstrual cycles progressed [4, 17–19]; address endometrial differences related to age [20] or the dysfunction of the WOI between patients with implantation failure and healthy controls [21]; and finally, correct for the menstrual cycle bias that can mask important biomarkers [22]. Notwithstanding, the candidate biomarkers of endometrial receptivity reported in each of the aforementioned studies overlap poorly [7]. Indeed, recent comparisons conducted by Sebastian-Leon and colleagues [7] found no congruities between 16 different reported gene lists of endometrial receptivity, and only fair to moderate functional agreements between some of the included signatures. Despite previous transcriptional regulation studies, there have been no holistic studies of this regulatory process, which robustly analyse the drivers of transcription across a higher number of studies (that each searched for endometrial receptivity biomarkers) and map the relative contributions of each type of regulator to endometrial progression regulation (Ovarian hormones [i.e., estrogen, progesterone], TFs, miRNAs).

Thus, the aim of this study was to utilize available genomic data regarding transcriptional regulators to identify overlapping mediators of endometrial transcriptional regulation (i.e., ovarian hormones, TFs, and miRNAs) and determine the relative contribution of each type of regulator among the previously reported gene signatures. These findings could help unveil the master regulators and principal type of endometrial regulation, paving the way for further research aimed at improving woman´s reproductive health.

## Methods

A detailed study design is depicted in Supplementary Fig. S1.

### Annotating gene lists associated with endometrial progression and function

Endometrial progression and implantation failure gene lists [7] were retrieved and updated using a public data repository (i.e., Gene Expression Omnibus (GEO)). Keywords used for the GEO search included: *endometrial receptivity, mid-secretory endometrium, RIF, recurrent implantation failure, endometrium, unexplained infertility,* and *implantation failure*. The resulting datasets were filtered by their publication date (i.e., from January 2018 to October 2020, to expand the systematic search of [7], number of samples (> 3 samples for each condition), and species (i.e., *Homo sapiens),* with no restrictions on publication language. The original signatures were then retrieved from their corresponding publications. The genes prioritized in the original publication were exclusively selected and their names were annotated with HUGO Gene Nomenclature Committee (HGNC) gene name using biomaRt R-package v.3.10 [23]. Each signature was labelled with the name of first author of the corresponding study. Our annotated gene lists were selected as representative signatures of endometrial progression and function for subsequent analyses (Supplementary Fig. S1A).

### Identifying hormonal and non-hormonal gene regulators of endometrial progression and function

To study the hormonal regulation of endometrial progression, the Kyoto Encyclopaedia of Genes and Genomes (KEGG) [24], and Gene Ontology (GO) [25] databases were consulted for ovarian hormone-related genes, using relevant keywords (i.e., *progesterone*, *estrogen*, *oestrogen* and *estradiol)* and selecting those pathways and functions that contained them (Supplementary Fig. S1B). Genes associated with the obtained human KEGG pathways and GO functions were grouped according to whether they were unique to progesterone (P4 gene set) or estrogen (E2 gene set) or related to both hormones (P4 and E2 gene set). Gene targets of the nuclear progesterone (*PGR*) and estrogen (*ESR1, ESR2)* receptors were added to their corresponding gene sets using DoRothEA (Discriminant Regulon Expression Analysis) database [26], considering only manually-curated or ChiP-Seq experimentally-validated gene-TF relationships. Finally, the P4 and E2 gene sets were independently mapped to each gene list.

Meanwhile, to evaluate the non-hormonal regulators of endometrial progression and function, DoRothEA [26] and TarBase [27] databases were consulted to obtain TFs and miRNAs. DoRothEA was filtered as previously described herein, while TarBase, which only contains miRNA-gene target relationships manually curated from publications or experimentally-validated in high-throughput datasets [27], was filtered by species (i.e., human). Finally, a functional over-representation analysis was carried out to identify which of the total annotated TFs or miRNAs were significantly associated with a particular gene list (Supplementary Fig. S1B).

To evaluate the relative contribution of each type of regulator (i.e., P4, E2, TFs, and miRNAs) on endometrial progression and function, three different comparisons were carried out applying Fisher’s exact tests. First, hormonal regulators were evaluated using the relative proportion of P4- and E2-related genes included in each list, with respect to the total of number of genes founded in the P4 or E2 gene sets. Then, non-hormonal regulators were evaluated using the relative proportion of over-represented miRNAs and TFs with respect to the total of miRNAs and TFs with a at least one target in the gene list. Finally, the relative proportion of genes under hormonal (i.e., P4 and/or E2-annotated genes) versus non-hormonal (i.e., miRNA and/or TF-annotated genes) regulation was evaluated considering the miRNAs/TFs found within the individual P4 or E2 gene sets as hormonal regulators.

### Prioritizing key regulators in endometrial progression and function

We built regulatory networks to systemically analyse endometrial regulation. Nodes represented gene lists and regulators (miRNAs or TFs), while edges indicated significant enrichment among the lists and their corresponding regulators (false discovery rate (FDR) ≤ 0.05). To select the most influential regulators (i.e., those which targeted genes in most of our signatures), we studied the degree distribution of the networks (i.e., the number of gene lists regulated by each molecule) and prioritized the miRNAs and TFs which surpassed the relative maximum (i.e., 1.50 times the interquartile range (IQR)) number of relationships (gene list–regulators). All networks were built and analysed with Cytoscape version 3.7 [28] (Supplementary Fig. S1B).

### Dataset construction and processing for in-silico validation

To evaluate how the miRNAs’ expression change in the endometrium throughout the menstrual cycle, we used the raw data from GSE44558 GEO dataset [29], deriving from 20 endometrial samples collected throughout the menstrual cycle: four in early-proliferative (EPF), four in late-proliferative (LPF), four in early-secretory (ESE), four in mid-secretory (MSE) and four in late-secretory (LSE).

To evaluate how the TFs’ expression change in the endometrium throughout the menstrual cycle, we analysed the integrated endometrial dataset previously created by our group, and reported in [30], which compiled data from five prior publications (GSE98386, GSE29981, GSE4888, GSE119209 and GSE86491). This dataset included endometrial gene expression data from 109 participants with normal endometrium, where biopsies were collected in proliferative (PF) (*n* = 29), ESE (*n* = 29), MSE (*n* = 43) and LSE (*n* = 8) phases of the menstrual cycle (Table S1).

Both TF and miRNA datasets were processed using the limma R-package [31]. Expression data were log transformed and quantile normalized, prior to exploratory analyses which sought out possible outliers and batch effects. Relative gene expression ranges of low (1–10%), medium (11–50%), and high (51–100%) were established based on the expression of all the genes included in our dataset.

### Wet lab validation cohorts

To corroborate the menstrual cycle-related expression changes observed with CTCF and GATA6 in our *in-silico* analysis, we conducted a prospective study as an external validation, using an independent sample set. We included a cohort of 19 healthy Spanish women (obtaining a total of 20 biopsies), between the ages of 22–35 and with a body mass index of 22.80 ± 2.76 kg/m^2^. Endometrial samples were collected and staged in the menstrual cycle according to the follicle growth to control ovulation and LH levels in urine. Samples were grouped into PF (*n* = 5), ESE (*n* = 5), MSE (*n* = 5) and LSE (*n* = 5) phases and categorized into two groups, PF/ESE and MSE/LSE.

### RNA extraction and RT-qPCR

Total RNA was extracted from the endometrial biopsy samples using the miRNeasy Mini Kit (Qiagen, Germany), and reverse transcribed into cDNA using the PrimeScript reagent kit (TAKARA, Japan). RT-qPCR reactions were carried out in duplicate, using fluorescent Power-up SYBR Green (Thermo Fisher Scientific MA, USA) in a final volume of 10 µL. Primer sequences are shown in Table S2. Samples underwent 40 cycles of amplification, under standard conditions, using a StepOnePlus™ System (Applied Biosystems, MA, USA). Relative mRNA expression was calculated using the 2^−ΔΔCt^ method [32], and normalized to the expression of GAPDH housekeeping gene.

### Statistical analysis

Fisher’s exact test was used to evaluate independent proportions. Mean expression changes across the cycle were studied using an analysis of variance (ANOVA) followed by a pairwise t-test or the Wilcoxon test when only two groups were available. *P*-values from multiple test comparisons were corrected by FDR and considered as significant when FDR ≤ 0.05. All statistical analyses were performed through R software (version 3.5) [33].

## Results

### Gene signatures associated to endometrial progression and function

Of the 19 gene lists used for our analysis, eleven were obtained from published studies evaluating control patients throughout the menstrual cycle [4, 17–19, 34–40], and the other eight were derived from studies comparing patients with RIF or unexplained infertility to controls [21, 41–47] (Table 1). Unifying all the aforementioned signatures, we compiled 3,608 genes related to endometrial progression and function.

**Table 1:** Characteristics of the 19 gene lists included in this study. Gene lists were divided according to their evaluated condition. Each signature was labelled using the name of first author, followed by the publication year in the case of duplicates. Endometrial staging was based on different variables including urinary LH, histological analysis, ultrasound evaluation, or the number of days since the hCG trigger. Participants included healthy controls and women with infertility-related conditions. Various platforms were used to evaluate gene expression. The thresholds applied for gene refinement and the final number of genes per signature are also presented

**Condition**	**Gene signature**	**Endometrial staging**	**Participants**	**Platform**	**Threshold**	**No. genes**	**References**
Endometrial progression	Altmäe2017	N/A	N/A	N/A	RRA	57	[34]
	Borthwick	Urinary LH; Histologically confirmed	PF d9-11 (*n* = 5) vs R LH + 6–8 (*n* = 5)	Affymetrix Genechip Hu95A	N/A	116	[18]
	Carrascosa	N/A	N/A	Quantitative RT-PCR	Based on literature	187	[35]
	Carson	Urinary LH; Histologically confirmed	ESE LH + 2–4 (*n* = 3) vs MSE LH + 7–9 (*n* = 3)	Affymetrix Genechip Hu95A	|FC|> 2	695	[17]
	Diaz-Gimeno	Urinary LH	PR LH + 1–5 (*n* = 15) vs R	Agilent custom gene expression microarray	|FC| > 2	234	[36]
	Kao	Urinary LH; Histologically confirmed	LPF d8-10 (*n* = 4) vs MSE LH + 8-10 (*n* = 7)	Affymetrix Genechip Hu95A	|FC|> 2 and *P*-value <0.05	340	[37]
	Mirkin	Urinary LH; Histological dating	ESE LH + 3 (*n* = 3) vs MSE LH + 8 (*n* = 5)	Affymetrix Genechip Hu95A	|FC|>2 and adj-*P*-value < 0.05	105	[19]
	Ponnampalam	Histological dating	EPF (*n* = 5) MPF (*n* = 7) LPF (*n* = 3) ESE (*n* = 7) MSE (*n* = 8) LS (*n* = 7) Mense (*n* = 6)	Custom	Adj-*P*-value <0.05	306	[38]
	Punyadeera	Histological dating	Mense (*n* = 2) vs LPF (*n*=2)	Affymetrix HG-U133A	FC and *P*-value	50	[39]
	Riesewijk	Urinary LH; Histological dating	PR LH + 2 (*n* = 5) vs R LH + 7 (*n* = 5)	Affymetrix Genechip Hu95A	|FC|> 3 in at least four out of five women.	196	[40]
	Talbi	Histological dating	P (*n* = 6) vs ESE (*n *= 3) vs MSE (*n* = 8) vs LSE (*n* = 6)	Affymetrix HG-U133 Plus 2.0	|FC|> 1.5 and adj-*P*-value < 0.05	317	[4]
Implantation failure	Altmäe2010	Urinary LH	UI (*n* = 4) vs C (*n* = 5)	Whole Human Genome Oligo Microarray (Agilent Technologies)	|FC|> 2 and PFP < 0.05	184	[21]
	Bastu	6-7 days after LH surge	IF (*n* = 24) vs Fertile women (*n* = 24)	Agilent-039494 SurePrint G3 Human GE v2 8x60K	Log2(FC) ≥ 1 and adj-*P*-value ≤0.05 |FC|> 2 and PFP < 0.05	524	[41]
	Bersinger	N/A	IF (*n* = 3) vs M (*n* = 3) vs OP (*n* = 3)	Affymetrix Human Genome U133A 2.0 Chips	Not specified	93	[42]
	Bhagwat	N/A	PR vs R	Multiple platforms	Based on literature	177	[43]
	Koot	Urinary LH	RIF (*n *= 43) vs C (*n *= 72)	Human whole genome gene expression microarrays V2 (Agilent, Belgium)	Machine learning predictor	310	[44]
	Lédée	Ultrasound evaluation	IF (n = 30) vs Fertile women (*n* = 15)	Affymetrix Human Genome U133 Plus 2.0 Array	Adj-*P*-value <0.01 and Δ > 1.35	322	[45]
	Pathare	6-7 days after hCG administration	IF (*n* = 10) vs Healthy oocyte donors (*n* = 8)	Illumina HumanHT-12 V4.0 expression beadchip	|FC|> 2	818	[46]
	Shi	Urinary LH; Ultrasound evaluation	RIF (*n* = 12) vs C (*n* = 10)	Agilent-052909 CBC lncRNAmRNA V3	|FC|≥ 2 and adj-*P*-value ≤ 0.05	281	[47]

*Abbreviations*: *Δ* delta C_t_, *adj-P-value* Adjusted *P*-value, *C* Control group, *d* Day of menstrual cycle, *EPF* Early proliferative, *ESE* Early secretory, *|FC|* Absolute fold-change, *FC* Fold change, *HRT* Hormone replacement therapy, *IF* Implantation failure, *LH* Luteinizing hormone, *LH + X* X days post LH surge, *LPF* Late proliferative, *LSE* Late-secretory, *M* Miscarriage, *MPF* Mid-proliferative, *MSE* Mid-secretory, *n* number of samples, *N/A* Not applicable, *OP* Ongoing pregnancy, *PF* Proliferative, *PFP* Proportion of false positives, *PR* Pre-receptive, *R* Receptive, *RIF* Recurrent implantation failure, *RRA* Robust Rank Aggregation algorithm, *SF* Spontaneously fertile women, *UI* Women with unexplained infertility, *vs* versus

### Hormonal regulation of endometrial progression is largely driven by progesterone

**Fig. 1:**
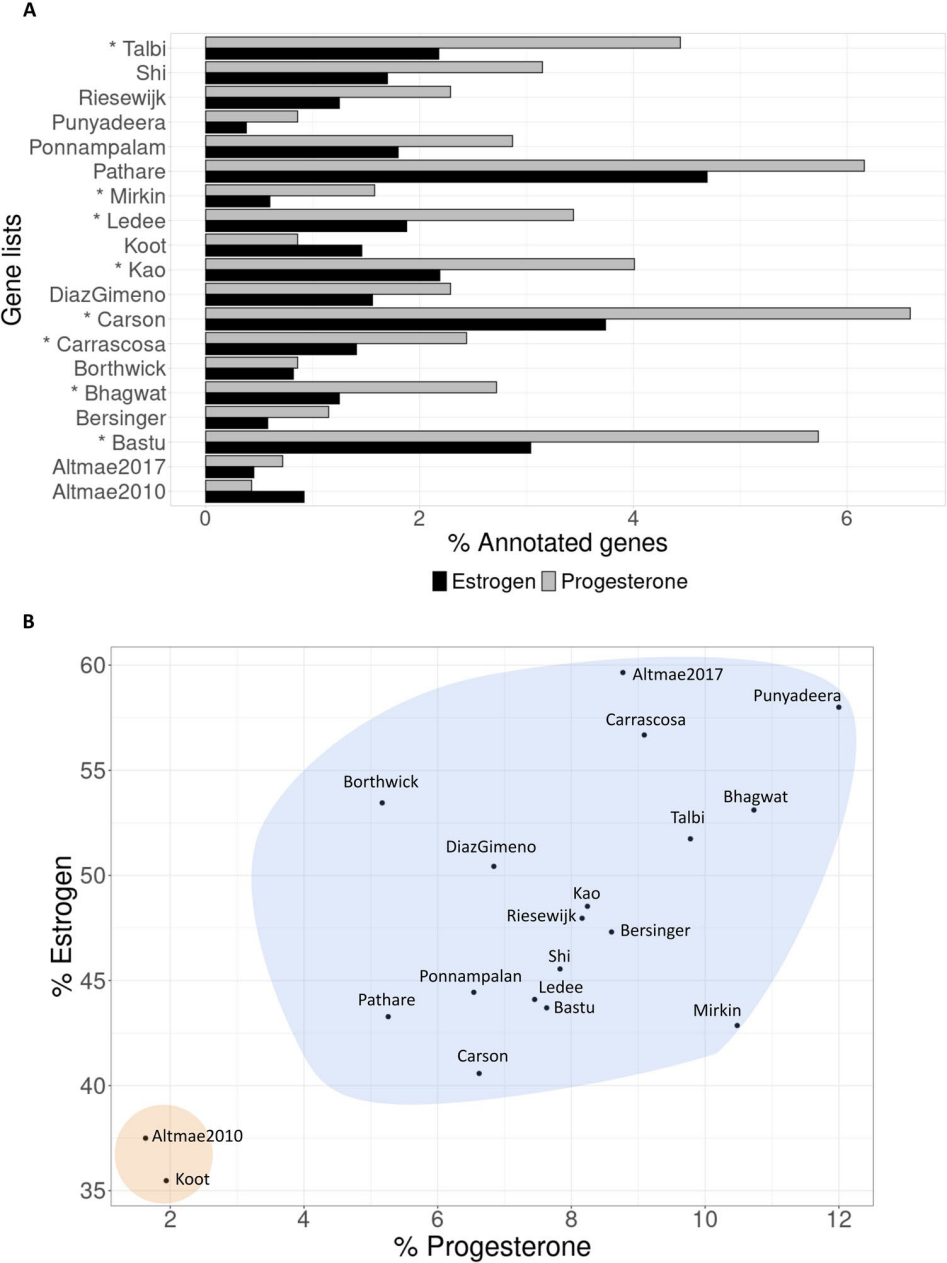
Estrogen and progesterone-mediated regulation of endometrial progression and function. **A** Eight out of nineteen gene lists (47%) had significant differences between the proportion of genes regulated by progesterone versus estrogen. (*FDR ≤ 0.05). **B** Descriptive analysis of hormonal regulation, normalized by the size of each gene list. Gene lists clustered according to higher (blue) or lower (orange) contribution of hormonal regulation

We identified 7,540 and 698 genes related to estrogen and progesterone hormones, respectively. However, as determined by the relative contribution of each type of hormone within each gene list (Fig. 1A), 17/19 (89%) signatures favoured regulation by progesterone rather than estrogen. These differences were significant in 47% of the signatures (FDR < 0.05). Exceptionally, the genes reported by the Altmae2010 and Koot signatures, that were related to unexplained infertility and RIF, had a preference for estrogen regulation (Fig. 1A). With respect to the proportion of progesterone-related genes, Altmae2010 showed 2.1 times more estrogen-related genes while Koot showed 1.7 times more. Moreover, comparing the proportion of genes regulated by both hormones, between signatures revealed that Altmae2010 and Koot exhibited a lower hormone-dependent behaviour overall, with respect to the remaining signatures (Fig. 1B), highlighting the role of non-hormonal regulators.

### Non-hormonal regulation of endometrial progression and function

We identified a total of 770 miRNAs and 555 TFs regulating at least one of the 3,608 genes reported in the 19 gene lists. Further, we observed that 29% of the miRNAs and 44% of the TFs were associated with the ovarian hormones (i.e., progesterone and/or estrogen). Significantly more genes were targeted by hormonally-influenced regulators rather than non-hormonal regulators (*P*-value < 0.01, odds ratio = 91.94). Nevertheless, we also identified 595 miRNAs (77.27%) and 311 TFs (56.04%) that were unrelated to progesterone and/or estrogen, highlighting a substantial proportion of hormonally-independent regulators of the menstrual cycle.

Of the respective miRNAs and TFs targeting genes related to endometrial progression and function, 417 miRNAs and 467 TFs were significantly over-represented in at least one gene list (FDR < 0.05, Tables S3-S4). Further, most signatures were significantly governed by TFs rather than miRNAs (4 > odds ratio > 164, FDR < 0.05, 17 lists [89%]), with the exception of Koot’s signature that was governed by miRNAs and showed the opposite (odds ratio = 0.35, FDR < 0.05, Table S5).

### Hsa-miR-15a-5p, hsa-miR-128-3p, hsa-miR-218-5p, hsa-miR-27b-3p, hsa-miR-107, hsa-miR-424-5p, hsa-miR-195-5p, hsa-miR-103a-3p, has-let-7b-5p and hsa-miR-22-3p are key players in the regulation of endometrial progression and function

**Fig. 2:**
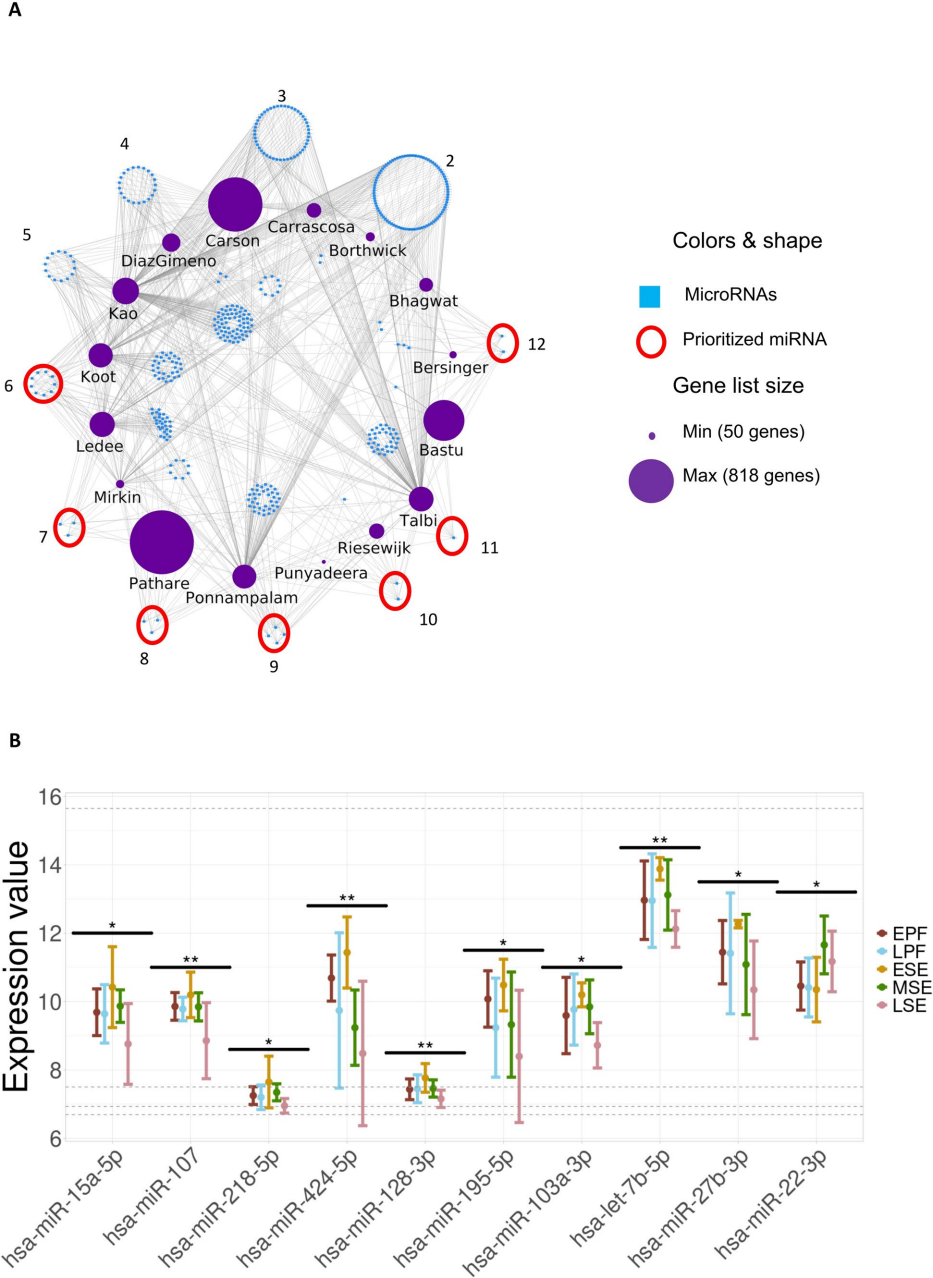
MicroRNAs universally regulating endometrial progression and function and their expression throughout the menstrual cycle. **A** Regulatory network of miRNAs forming relationships with numerous gene lists (2–12); miRNAs targeting specific gene lists were placed in the centre of the network. The prioritized miRNAs (outlined in red) included has-miR-16-5p, has-miR-138-5p, has-miR-21-3p, has-miR-205-5p, has-miR-27a-5p, has-miR-15a-5p, has-miR-155-5p, has-miR-129–2-3p, has-miR-147a, has-miR-146a-5p, has-miR-107, has-miR-424-5p, has-miR-203a-3p, has-miR-195-5p, has-miR-124-3p, has-miR-128-3p, has-miR-22-3p, has-miR-27b-3p, has-let-7b-5p, has-miR-1343-3p, has-miR-1-3p, has-miR-103a-3p, has-miR-23b-3p, has-miR-218-5p. **B** Expression profiles of the 10 prioritized miRNAs at different phases of the menstrual cycle. Significant changes between phases are denoted with asterisks (* FDR ≤ 0.05, ** FDR ≤ 0.01. Abbreviations: EPF, early proliferative phase; LPF, late proliferative phase; ESE, early-secretory phase; MSE, mid-secretory phase; LSE, late-secretory phase

To bridge the gap of endometrial-based evidence in DoRothEA and TarBase databases, we aimed to identify the common miRNAs that mediate gene expression related to endometrial progression and function. We evaluated the number of endometrial gene lists that each miRNA controlled (Fig. 2A). This strategy highlighted the most influential miRNAs that were commonly found across the endometrial gene lists. Notably, 85.80% of miRNAs regulated genes from one to three signatures, and only 59 miRNAs (14.20%) were associated with more than three signatures**.** Ultimately, 24 miRNAs were prioritized for regulating genes of at least six signatures.

Next, we validated the gene expression trends for these 24 prioritized miRNAs in an independent endometrial gene expression dataset (GSE44730) (Fig. 2B). This dataset included the miRNA expression in endometrial biopsies from 20 healthy women in different phases of the menstrual cycle. We found that two of our prioritized miRNAs were not expressed in the endometrial tissue, and only 10 miRNAs (i.e., hsa-miR-15a-5p, hsa-miR-128-3p, hsa-miR-218-5p, hsa-miR-27b-3p, hsa-miR-107, hsa-miR-424-5p, hsa-miR-195-5p, hsa-miR-103a-3p, has-let-7b-5p and hsa-miR-22-3p) showed significant changes across the menstrual cycle (Fig. 2B). Of these, only the expression of hsa-miR-22-3p significantly increased from ESE to MSE stages (FDR = 0.02, Fig. 2B), highlighting the potential inhibitory role of this miRNA in endometrial function. The expression of the remaining miRNAs decreased during the MSE phase.

### CTCF and GATA6 as distinguished transcriptional regulators of the human menstrual cycle

**Fig. 3:**
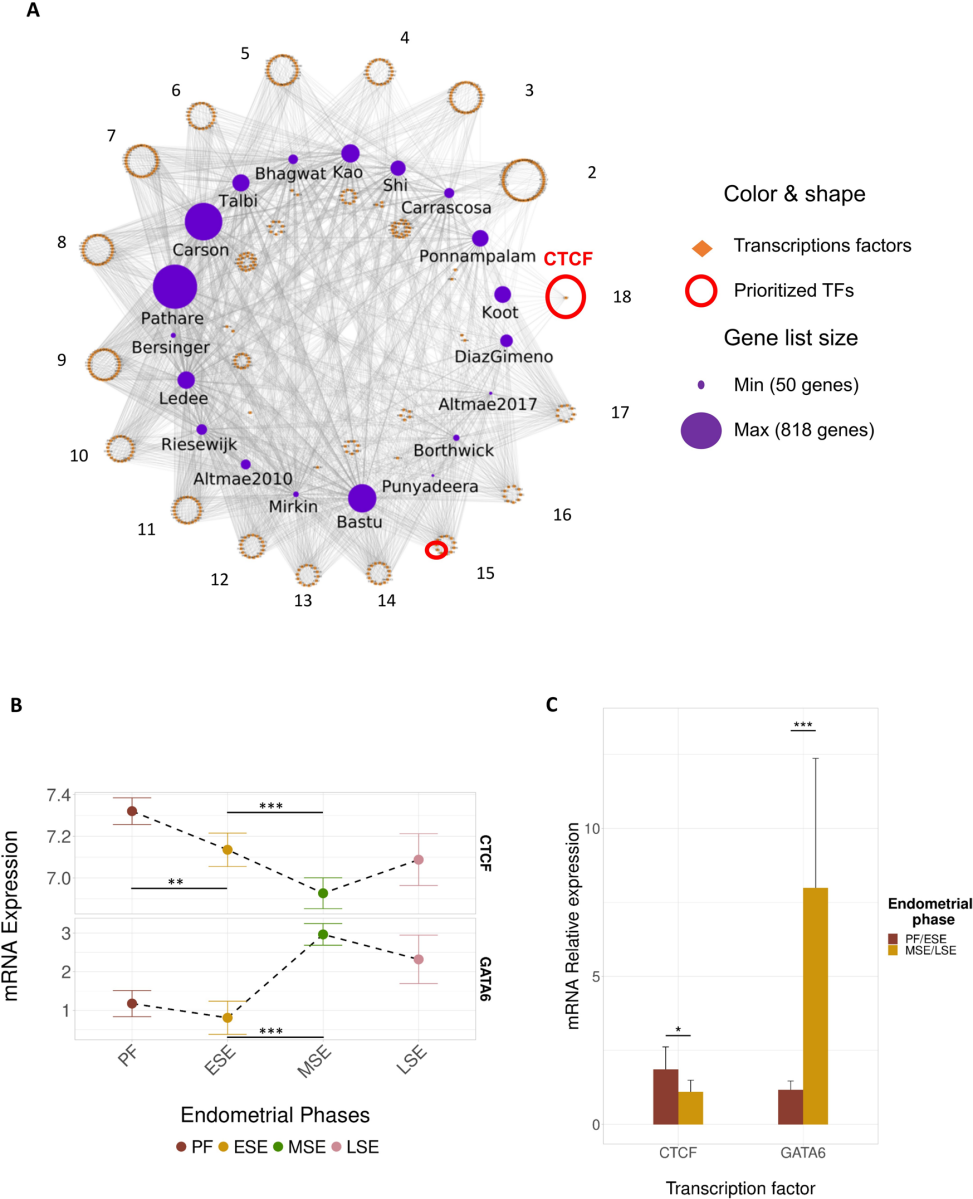
Transcription factors universally regulating endometrial progression and function and their expression throughout the menstrual cycle. **A** Regulatory network of TFs forming relationships with numerous gene lists (2–18). TFs targeting specific gene lists were placed in the center of the network. *CTCF* stood out as the only TF that targeted genes belonging to 18/19 (95%) different signatures. **B** *In-silico* analysis of *CTCF* and GATA6 expression throughout the menstrual cycle. Significant changes between phases are marked with asterisks (**FDR ≤ 0.01, ***FDR ≤ 0.001). **C** RT-qPCR validation. Bar plot shows the relative mRNA expression of CTCF and GATA6 in endometrial biopsies collected throughout the menstrual cycle. Data is presented as a mean ± SD. Significant differences of CTCF (*FDR < 0.05) and GATA6 (***FDR < 0.001) expression were observed between proliferative/early-secretory (PF/ESE) and mid-secretory/late-secretory phases (MSE/LSE). Abbreviations in (B and C): PF, Proliferative; ESE, early-secretory; MSE, mid-secretory; LSE, late-secretory; SD, standard deviation

We repeated the regulation network analysis with the TFs, to identify the prominent TFs commonly regulating the genes related to endometrial progression and function in the reported signatures. We observed 78.80% of the TFs regulated genes in up to 10 signatures, and only 99 TFs (21.20%) regulated genes in over 10 (Fig. 3A). We highlighted *CTCF* (a CCCTC-binding factor, which functions as a transcriptomic repressor) as the TF with the most influential regulation of endometrial progression and function across 95% of studies (18 gene lists, excluding that of Punyadeera, Fig. 3A). Other distinguished TFs that regulated up to 15 gene lists included *AR, CEBPA, CEBPB, CEBPD, CREB1, EGR1, ELF3, ESR1, ETV4, FOS, FOXA1, GATA2, GATA3, GATA6, HNF4A, JUN, JUND, NFKB1, NR1H2, NR3C1, RELA, SMAD3, SP1, SPI1, STAT1, STAT3, TCF7L2, TFAP2C* and *TP53.* Notably, GATA6 was selected due to novel association with endometrial progression and function.

To infer the expression of *CTCF* and *GATA6* in endometrial tissue throughout the menstrual cycle, we analysed their expression using a relevant endometrial dataset recently created by our group [30] (Table S1). We observed that, relative to the global expression of endometrial genes, *CTCF* was broadly expressed while *GATA6* was moderately expressed. Indeed, *CTCF* was significantly down-regulated between the PF and ESE phases (FDR = 2.10E-03) and ESE to the MSE phase (FDR = 2.10E-04), while GATA6 expression increased from the PF to the MSE phase (FDR = 1.70E-11) and from the ESE to MSE phase (FDR = 9.80E-15). These findings supported the role of these TFs at the beginning of the menstrual cycle and during the MSE phase (Fig. 3B).

These *in-silico* validations were then prospectively corroborated by RT-qPCR analysis in an independent sample set of 20 endometrial biopsies. We observed a similar expression profile of *CTCF* and *GATA6* throughout the menstrual cycle, with significant changes between the endometrial phases. Notably, *CTCF* expression was decreased between the PF/ESE and MSE/LSE phases (FDR = 0.01) while GATA6 expression increased from the PF/ESE to MSE/LSE phases (FDR = 1.55E-04) (Fig. 3C).

## Discussion

This data-driven approach exposed the common transcriptional regulators among 19 studies who proposed variable biomarkers of endometrial progression and function. In this study, we focused on understanding the relative contribution of both the hormonal and non-hormonal regulation, from an alternative holistic perspective. We applied data-driven hypothesis research that, unlike the traditional scientific method, allowed us to generate new hypotheses based on all available biologically-relevant knowledge [16], observing the molecular relationships from a wider scale view. Besides, some traditional molecular procedures such as PCRs are also performed to corroborate the insight uncovered with this approach. We highlighted a larger influence of progesterone-related genes and TFs in endometrial progression and observed a larger contribution of miRNAs and estrogen-related genes in endometrial pathology. Furthermore, we unveiled CTCF, GATA6, hsa-miR-15a-5p, hsa-miR-218-5p, hsa-miR-107, hsa-miR-103a-3p and hsa-miR-128-3p as master regulators in the endometrium, and these findings were reinforced by the dynamic changes in their expression throughout the menstrual cycle (dually validated with *in-silico* and prospective analyses).

The menstrual cycle is perceived to be under tight regulation by the ovarian hormones, progesterone and estrogen [48]. Supporting the role of progesterone in the secretory endometrium [49], and reinforcing its indispensable role in endometrial regulation [50, 51], we found progesterone and its related genes had a larger influence in menstrual cycle progression than estrogen. As the RIF-related gene lists reported by Altmae2010 and Koot were generally controlled by estrogen, this further suggested that while progesterone could be crucial for a suitable endometrial progression and mid-secretory phase acquisition, estrogenic irregularities may be more conducive to endometrial function and molecular pathology. Indeed, this hypothesis corroborated previous reports of most uterine disorders being estrogen-dependent [44, 52]. Supporting the findings from another recent study from our group, where the Altmae2010 and Koot signatures were classified as highly-predictive signatures to identify endometrial pathology in comparison with a prediction model that detected endometrial progression [7]. This study distinguished these signatures (related to implantation failure and unexplained infertility) based on the magnitude of their miRNA and estrogen-regulation, which contrasted with the prominent influence of the TFs over the remaining signatures more related to endometrial progression. All these facts reinforce the hypothesis that implantation failure is mainly caused by impaired miRNA expression [53].

Although we observed a clear hormonal influence in numerous annotated regulators, this study transcended the simplistic paradigm of the bihormonal regulation of endometrial function by identifying a plethora of mediators, including 595 miRNAs and 311 TFs, that were previously unrelated to these ovarian hormones. These findings set the foundation for new discoveries, revealing alternative pathways and new actors in the regulation of the human endometrium, and deepening our understanding of the complex regulatory mechanisms behind endometrial progression and unexplained infertility.

MicroRNAs are well known to be involved in embryo implantation [12] and receptivity control [54, 55]. Using network analysis, we unveiled the 10 common miRNAs regulating endometrial progression and function across published biomarker signatures (hsa-miR-22-3p, hsa-miR-107, hsa-miR-103a-3p, hsa-miR-128-3p, hsa-miR-195-5p, hsa-miR-218-5p, hsa-miR-15a-5p, hsa-miR-27b-3p, hsa-miR-424-5p, and has-let-7b-5p). Their significant gene expression changes in the secretory endometrium support their potential role in the endometrial regulation through an inhibition during the WOI. Notably, hsa-miR-424-5p, hsa-miR-27b-3p and hsa-miR-195-5p have been previously associated with RIF [11, 54, 56], while has-let-7b-5p was associated with human endometrial receptivity [57], and hsa-miR-22-3p was overexpressed in a decidualization model [58]. Moreover, hsa-miR-15a-5p was previously associated with endometriosis [59]. Notwithstanding, this study is the first to associate hsa-miR-15a-5p, hsa-miR-218-5p, hsa-miR-103a-3p, hsa-miR-107, and hsa-miR-128-3p to endometrial progression and functional regulation in humans.

Due to their direct relationship with progesterone and estrogen [60], we expected to find TFs, such as *ESR1* and *PGR,* involved in endometrial progression, however, we additionally identified other TFs with an even larger influence in this process. For instance, *STAT3* was associated with 17/19 signatures (FDR < 0.05), and is known to regulate uterine epithelial junctional reorganization and stromal proliferation, which are critical for implantation [61]. *SP1*, which similarly regulated 17/19 signatures (FDR < 0.05), acts as a downstream paracrine target of progesterone to regulate estrogen inactivation, and could have a predominant role during the WOI [62, 63]. By focusing on the most universal, overlapping TFs that were not previously related to implantation, we ultimately prioritized *CTCF* and *GATA6* as novel key regulators of endometrial progression, however, their specific molecular actions in endometrial progression and function were beyond the scope of this project and merit further investigation.

*CTCF* is a conserved zinc finger protein whose regulatory functions are well characterized throughout the human body [64]. *CTCF* acts as a transcriptional repressor in RNA polymerase II (Pol II) pausing and imprinting and X-chromosome inactivation [65], as well as an insulator, blocking the interaction between enhancers and the promoters of neighbouring genes [66]. Considering these attributed functions and its variable expression throughout the menstrual cycle, we propose that *CTCF* exerts an inhibitory role in endometrial tissue during the PF phase of the menstrual cycle. With the significant downregulation of *CTCF* in the secretory endometrium, dually validated *in-silico* and experimentally herein, its inhibited genes would be derepressed and become transcriptionally active during the WOI. This interpretation supports previous findings from our group demonstrating that, during the WOI, a global transcriptional derepression may be required for implantation and early embryo development [63]. Transcriptional derepression has been associated with multiple human disease states and should be investigated further within the context of endometrial-factor infertility. Despite previous associations of *CTCF* with endometriosis [67], its implication in endometrial progression and function has not been directly proposed until now. Nevertheless, the role of CTCF in endometrial receptivity has been revealed through its interaction with HOXA10, a gene mainly expressed in endometrium involved in functions such as endometrial proliferation and differentiation, the formation of pinopodes or embryo implantation [68]. Indeed, this study proposed that an overexpression of CTCF can lead to a drop in HOXA10 expression, affecting endometrial proliferation and endometrial function and corroborating the fact that CTCF must decrease its expression as we can observe in our findings.

GATA6, the second prioritized TF that overlapped across the gene lists, is a transcription factor belonging to the GATA family, a highly conserved family of six zinc finger proteins [69]. Besides its essential role in embryonic development [70], GATA6 plays a key role in endometriosis and regulates steroidogenic genes [71]. Together with evidence supporting both an activating and inhibitory role for GATA6 [72, 73], the significant changes we observed in its expression across the menstrual cycle reflect its presumed involvement in the endometrium and suggest an activating role in endometrial progression and endometrial receptivity acquisition. Like CTCF, this is the first time that GATA6 is proposed as a key TF in endometrial progression and function.

Although the exact molecular mechanisms underlying endometrial regulation remain elusive, emerging experimental technologies such as High-throughput Chromosome Conformation Capture (Hi-C) or chromatin interaction analysis with paired-end tags (ChIA-PET) that respectively study general chromatin interactions or chromatin interactions involving a specific protein [74, 75] may improve our understanding of genetic regulation in the future. Nevertheless, as collaborative efforts unveil new regulators and gene targets in humans (such as the ENCODE project [76]), the complexities of transcriptional regulation can be exposed by *in-silico* analyses like those described herein, and we can gain a better understanding of the processes involved in menstrual cycle progression and endometrial competence.

Furthermore, it should be noted that results of gene expression studies are influenced by different variables, such as the experimental platforms employed, study designs, or patients’ characteristics of each independent study, adding variability [30, 77, 78]. Despite this fact, we have found that our holistic approach overcomes this undesired variability effect by finding common upstream master regulators across studies and predicting new relationships between hormones, TFs and miRNAs. This approach also provides the basis for future single-molecule studies that aim to elucidate new regulatory pathways in endometrial progression and function. Our results lay the foundation for further molecular studies that can validate the function(s) of the prevailing endometrial regulators we prioritized.

## Conclusion

The study shows for the first time, the relative contribution of estrogens, progesterone, TFs and miRNAs in endometrial function and progression. Endometrial progression is mainly influenced by progesterone-related genes and TFs, whereas miRNAs and estrogen-related genes play a larger role in endometrial pathology. Moreover, we highlight novel common transcriptional regulators such as CTCF, GATA6, hsa-miR-15a-5p, hsa-miR-218-5p, hsa-miR-107, hsa-miR-103a-3p, and hsa-miR-128-3p across 19 studies that propose biomarkers in endometrial regulation. These results reveal the molecular mechanism underlying endometrial regulation and lay the foundation for the development of targeted therapies for patients with endometrial-factor infertility.

### Supplementary Information

**Additional file 1: Supplementary Figure S1.** Study design and workflow. Steps performed throughout the study. (A) We reviewed 19 publications related to endometrial progression and function, extracted the gene signatures from the original publication, and created corresponding gene lists that we could annotate. (B) We performed different functional analyses based on the type of regulator (i.e., ovarian hormones, transcription factors and miRNAs). We used online databases to obtain the information of the regulatory process, at the hormonal (Gene Ontology, Kyoto Encyclopedia of Genes and Genomes, Dorothea), transcription factor (Dorothea) and miRNA (Tarbase) level and analysed the results accordingly. Finally, we employed an integrative analysis and prioritized the most relevant regulators for subsequent validation. TF, Transcription factors; miRNA, microRNA.

**Additional file 2: Supplementary Table S1.** Characteristics of endometrial transcriptomic datasets used to build the integrated dataset. ID, identifier of Gene Expression Omnibus database; Source, first author and year of the publication if it is available; No. samples; number of samples included; Age, range of patient age; Participants, reason for endometrial sample collection; Cycle Type, type of menstrual cycle of subjects included; Platform, platform used to measure gene expression; No. genes, number of genes measured in each dataset, Endometrial phase, number of samples in each phase of menstrual cycle. PF, proliferative; ESE, early secretory; MSE, mid secretory, LSE, late secretory. NA, Not Available.

**Additional file 3: Supplementary Table S2.** Primer sequences for CTCF and GATA6. Forward and reverse primers used for RT-qPCR validation of CTCF and GATA6 transcription factors.

**Additional file 4: Supplementary** **Table S3.** Results of miRNA enrichment. Excel file with the over-representation analysis results of the microRNAs (miRNAs). Each sheet contains the results of a specific list of genes. In each sheet, the following information is included: ID, gene symbol ID of the miRNA; GeneRatio, the proportion of miRNA targets in the gene list; BgRatio, the proportion of miRNA targets in the whole genome; OddsRatio; the odd ratios for each miRNA, pvalue; the *P*-value obtained for each miRNA; Padjust, *P*-value adjusted by FDR for each miRNA; geneID, gene symbol ID of the miRNA targets.

**Additional file 5: Supplementary** **Table S4.** Results of TFs enrichment. Excel file with the over-representation analysis results of the transcription factors (TFs). Each sheet contains the results of a specific list of genes. In each sheet, the following information is included: ID, gene symbol of the TF; GeneRatio, the proportion of TF targets in the gene list; BgRatio, the proportion of TF targets in the whole genome; OddsRatio; the odd ratios for each TF, pvalue; the *P*-value obtained for each TF; padjust, *P*-value adjusted by FDR for each TF; geneID, the gene symbol ID of the TF targets.

**Additional file 6: Supplementary** **Table S5.** Relative contribution of each regulator type (TFs or miRNAs) in each gene list. A Fisher’s test was performed to calculate the proportional differences between transcription factors (TFs) and microRNAs (miRNAs). Resulting *P*-values were adjusted by False Discovery Rate (FDR). Odds Ratios > 1 indicate gene lists were mainly regulated by TFs, whereas odds ratios < 1 indicate a higher regulation by miRNAs. Odds Ratio with a value of “Inf” indicate that miRNAs were not found for this gene list. Percentages indicate the proportion between the significant regulators in each gene signature and regulators with at least one target gene in the corresponding signature. (***FDR ≤0.001).

## Data Availability

The data used to analyze the TF and miRNA expression throughout the menstrual cycle and in infertility are available in Gene Expression Omnibus, with IDs GSE98386, GSE29981, GSE4888, GSE119209, GSE86491, and GSE44558.

## Abbreviations

ANOVA: Analysis of variance
ChIA-PET: Chromatin interaction analysis with paired-end tags
EPF: Early proliferative
ESE: Early secretory
FDR: False Discovery Rate
GEO: Gene Expression Omnibus
GO: Gene Ontology
HGNC: HUGO Gene Nomenclature Committee
Hi-C: High-throughput Chromosome Conformation Capture
IQR: Interquartile range
KEGG: Kyoto Encyclopaedia of Genes and Genomes
miRNAs: MicroRNAs
MSE: Mid Secretory
LPF: Late proliferative
LSE: Late secretory
RIF: Recurrent Implantation Failure
Pol II: RNA polymerase II
TFs: Transcription Factors
WOI: Window of implantation
