# Supplementary figures and images for: Deciphering a shared transcriptomic regulation and the relative contribution of each regulator type through endometrial gene expression signatures
Source: Reprod Biol Endocrinol. 2023 Sep 12;21:84. doi: 10.1186/s12958-023-01131-4 (PMC10496172; doi:10.1186/s12958-023-01131-4)

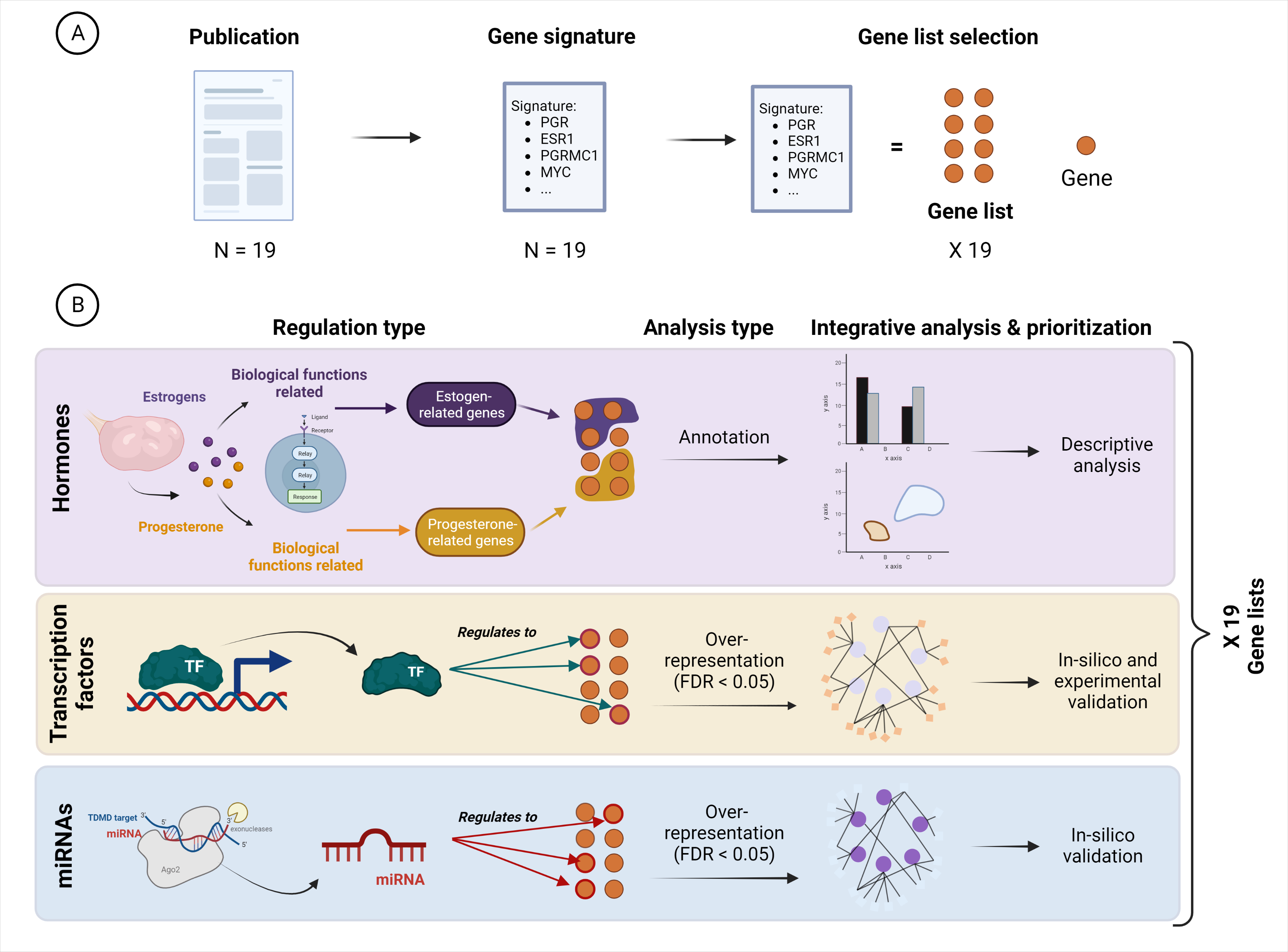

Supplement: Supplementary file 1 — Additional file 1: Supplementary Figure S1. Study design and workflow. Steps performed throughout the study. (A) We reviewed 19 publications related to endometrial progression and function, extracted the gene signatures from the original publication, and created corresponding gene lists that we could annotate. (B) We performed different functional analyses based on the type of regulator (i.e., ovarian hormones, transcription factors and miRNAs). We used online databases to obtain the information of the regulatory process, at the hormonal (Gene Ontology, Kyoto Encyclopedia of Genes and Genomes, Dorothea), transcription factor (Dorothea) and miRNA (Tarbase) level and analysed the results accordingly. Finally, we employed an integrative analysis and prioritized the most relevant regulators for subsequent validation. TF, Transcription factors; miRNA, microRNA. [file 12958_2023_1131_MOESM1_ESM.jpg]
