# Supplementary material for: Deciphering a shared transcriptomic regulation and the relative contribution of each regulator type through endometrial gene expression signatures
Source: Reprod Biol Endocrinol. 2023 Sep 12;21:84. doi: 10.1186/s12958-023-01131-4 (PMC10496172; doi:10.1186/s12958-023-01131-4)
Supplement: Supplementary file 2 — Additional file 2: Supplementary Table S1. Characteristics of endometrial transcriptomic datasets used to build the integrated dataset. ID, identifier of Gene Expression Omnibus database; Source, first author and year of the publication if it is available; No. samples; number of samples included; Age, range of patient age; Participants, reason for endometrial sample collection; Cycle Type, type of menstrual cycle of subjects included; Platform, platform used to measure gene expression; No. genes, number of genes measured in each dataset, Endometrial phase, number of samples in each phase of menstrual cycle. PF, proliferative; ESE, early secretory; MSE, mid secretory, LSE, late secretory. NA, Not Available. [file 12958_2023_1131_MOESM2_ESM.docx]

| **ID** | **Source** | **No. Samples** | **Age** | **Participants** | **Cycle Type** | **Platform** | **No.genes** | **Endometrial phases** |
| --- | --- | --- | --- | --- | --- | --- | --- | --- |
| GSE4888 | Talbi *et al.*, 2006 | 27 | 23-50 | Patients  (n = 20); Healthy fertile women  (n = 7) | Natural cycle; no hormonal treatment in the last 3 months | Affymetrix | 19,361 | PF (n = 6);  ESE (n = 4);  MSE (n =9)  LSE (n=8) |
| GSE29981 | NA | 19 | 20-39 | NA | Regular cycle | Affymetrix | 19,361 | PF (n = 10);  ESE (n = 6);  MSE (n =3) |
| GSE119209 | NA | 11 | NA | NA | NA | Illumina | 17,934 | PF (n = 6);  MSE (n =5) |
| GSE98386 | Altmäe *et al.*, 2017 | 38 | NA | Healthy fertile women  (n = 19) | Natural cycle; no hormonal treatment in the last 3 months | Illumina | 16,426 | ESE (n = 19);  MSE (n =19) |
| GSE86491 | Sigurgeirsson *et al.*, 2017 | 14 | 24-30 | Healthy fertile women  (n = 7) | Natural cycle; no hormonal treatment in the last 3 months. | Illumina | 15,939 | PF (n = 7);  MSE (n = 7) |

**Table SI. Characteristics of endometrial transcriptomic datasets used to build the integrated dataset.** ID, identifier of Gene Expression Omnibus database; Source, first author and year of the publication if it is available; No. samples; number of samples included; Age, range of age; Participants, reason for endometrial sample collection; Cycle Type, type of menstrual cycle of subjects included; Platform, platform used to measure gene expression; No. genes, number of genes measured in each dataset, Endometrial phase, number of samples in each phase of menstrual cycle. PF, proliferative; ESE, early secretory; MSE, mid secretory, LSE, late secretory. NA, Not Available.
