# Supplementary material for: Deciphering a shared transcriptomic regulation and the relative contribution of each regulator type through endometrial gene expression signatures
Source: Reprod Biol Endocrinol. 2023 Sep 12;21:84. doi: 10.1186/s12958-023-01131-4 (PMC10496172; doi:10.1186/s12958-023-01131-4)
Supplement: Supplementary file 3 — Additional file 3: Supplementary Table S2. Primer sequences for CTCF and GATA6. Forward and reverse primers used for RT-qPCR validation of CTCF and GATA6 transcription factors. [file 12958_2023_1131_MOESM3_ESM.docx]

| **TF** | **Primers** | |
| --- | --- | --- |
|  | **Forward** | **Reverse** |
| **CTCF** | 5’-AACCAGCCCAAACAGAACCA-3’ | 3’-TCCTCTTCCTCTCCCTCTGC-5’ |
| **GATA6** | 5’-ACCACCTTATGGCGCAGAAA-3’ | 3’-ATAGCAAGTGGTCTGGGCAC -5’ |

**Table SII. Primer sequences for CTCF and GATA6.** Forward and reverse primers used in qPCR validation for transcription factors CTCF and GATA6.
