# Supplementary material for: Deciphering a shared transcriptomic regulation and the relative contribution of each regulator type through endometrial gene expression signatures
Source: Reprod Biol Endocrinol. 2023 Sep 12;21:84. doi: 10.1186/s12958-023-01131-4 (PMC10496172; doi:10.1186/s12958-023-01131-4)
Supplement: Supplementary file 6 — Additional file 6: Supplementary Table S5. Relative contribution of each regulator type (TFs or miRNAs) in each gene list. A Fisher’s test was performed to calculate the proportional differences between transcription factors (TFs) and microRNAs (miRNAs). Resulting P-values were adjusted by False Discovery Rate (FDR). Odds Ratios > 1 indicate gene lists were mainly regulated by TFs, whereas odds ratios < 1 indicate a higher regulation by miRNAs. Odds Ratio with a value of “Inf” indicate that miRNAs were not found for this gene list. Percentages indicate the proportion between the significant regulators in each gene signature and regulators with at least one target gene in the corresponding signature. (***FDR ≤0.001). [file 12958_2023_1131_MOESM6_ESM.docx]

| **Gene signatures** | **OddsRatio** | **% Enriched TFs** | **% Enriched miRNAs** | **FDR** |
| --- | --- | --- | --- | --- |
| Altmae2010 | NA | 0.32 % | 0.00% | 0.39 |
| Altmae2017 | NA | 28.98 % | 0.00 % | 2.26E-35 *** |
| Bastu | 112.96 | 63.41 % | 1.50 % | 5.72E-134 *** |
| Bersinger | 17.10 | 21.52 % | 1.57 % | 4.68E-23 *** |
| Bhagwat | 28.10 | 50.85 % | 3.54 % | 8.04E-74 *** |
| Borthwick | 37.58 | 32.46 % | 1.26 % | 3.29E-46 *** |
| Carrascosa | 74.13 | 54.04 % | 1.55 % | 1.29E-104 *** |
| Carson | 24.87 | 65.23 % | 6.99 % | 3.03E-114 *** |
| DiazGimeno | 7.92 | 21.97 % | 3.42 % | 6.02E-21 *** |
| Kao | 5.80 | 61.93 % | 21.89 % | 2.02E-42 *** |
| Koot | 0.35 | 4.49 % | 11.76 % | 7.64E-05 *** |
| Ledee | 25.80 | 74.81 % | 10.27 % | 3.08E-113 *** |
| Mirkin | 18.53 | 63.8 % | 8.65 % | 4.75E-78 *** |
| Pathare | 164.03 | 57.37 % | 0.81 % | 1.82E-124 *** |
| Ponnampalam | 4.01 | 38.35 % | 13.42 % | 1.38E-20 *** |
| Punyadeera | 15.55 | 9.48 % | 0.67 % | 2.48E-09 *** |
| Riesewijk | 75.33 | 52.65 % | 1.45 % | 6.57E-92 *** |
| Shi | NA | 49.42 % | 0.00 % | 3.89E-101 *** |
| Talbi | 4.79 | 51.76 % | 18.29 % | 7.35E-31 *** |

**Table SIV. Relative contribution of each regulator type (TFs or miRNAs) in each gene signature.** A Fisher’s test was performed to calculate the proportional differences between transcription factors (TFs) and microRNAs (miRNAs). Resulting p-values were adjusted by False Discovery Rate (FDR). Odds Ratios > 1 indicate gene signatures were mainly regulated by TFs, whereas odds ratios < 1 indicate a higher regulation by miRNAs. Odds Ratio with a value of “NA” indicate that miRNAs were not found for this gene signature. Percentages indicate the proportion between the significant regulators in each genes signature and regulators with at least a target gene in said signature. (***FDR ≤0.001).
